# Bioactive Compounds from Agrifood Byproducts: Their Use in Medicine and Biology

**DOI:** 10.3390/ijms25115776

**Published:** 2024-05-26

**Authors:** Paola Faraoni, Serena Laschi

**Affiliations:** 1 Department of Experimental and Clinic Biomedical Sciences “Mario Serio”, University of Florence, Viale Pieraccini 6, 50139 Florence, Italy; 2 “Nanobiosens” Joint Lab, University of Florence, Viale Pieraccini 6, 50139 Florence, Italy; serena.laschi@unifi.it; 3 Department of Chemistry “Ugo Schiff”, University of Florence Via Della Lastruccia 3, Sesto Fiorentino, 50019 Florence, Italy

Agrifood produces a high amount of waste, millions of tons per year worldwide, the disposal of which is a significant environmental, organizational, logistical, economic and ethic problem and in the last decades the scientific interest about this argument has increased significantly. In order to reduce this waste fits the circular economy, in which waste products represent secondary raw material that can be used for various purposes [1,2], as also specified in the European Union’s green goals for 2050 (European Green Deal) [3].

The waste from agrifood can generate many by-products that represent materials usable in various sectors, such as Biochar, a recycled carbonaceous material with multiple applications thanks to its adsorptive characteristics [4]. For instance, agrifood byproducts are used as fertilizers or insect repellents, for packaging and in bioconstruction, e.g., as varnish solvents, for food dyes, for energy production as biofuels and in biomedical field as bacterial culture media or histological solvents; as well as in cosmetics [5,6].

Moreover, many nutrients and bioactive compounds still remain in agrifood waste, from polysaccharides to carotenoids, vitamins, oligopeptides, pigments, and polyphenols. This abundance in bioactive compounds represents an important resource as these substances can have a beneficial effect on health due to their nutraceutical characteristics [7,8]. Interest in bioactive compounds is rising, in fact, the number of publications found in PubMed with the keyword “bioactive compound” increased from 3 in 1970 to 8636 in 2023.

The term “nutraceutical” was coined by Stephen De Felice in 1989, combining “nutrition” and “pharmaceutical”, and stands for a food or fraction thereof with positive health effects, ranging from the prevention to the treatment of a disease [9]. Nutraceuticals are, given their origin, generally non-hazardous, hypoallergenic and highly digestible substances. For this purpose, agrifood waste represents a remarkable resource both quantitatively and in terms of the variety of nutraceutical substances it contains. For substances of this kind there is increasing demand, in fact it is a topic felt by the population to be in good health by assuming substances of natural origin and perhaps decreasing the intake of drugs [8,10,11].

One aspect that should be considered is that such bioactive substances with benefits to health are obtained from the starting material by extraction and fermentation processes, the development of which requires extensive studies and tests, as well as using new technologies, with an approach that, while drawing on past experience, is modern. For this reason many investigations are performed in both the pharmaceutical and food sectors so that the processes that allow the recovery and concentration of these substances are not economically expensive and do not lead, in turn, to the production of high quantities of hazardous waste or with high environmental impact. To this aim, in this research area, there is an increasing effort towards the development of green and ecofriendly chemistry [6,7,12,13].

In addition, in agrifood waste (peels, leaves, seeds, flowers, fruits, and vegetables unsuitable for the market) the concentration of bioactive molecules is very high. An example is the olive pomace, a byproduct of virgin olive oil production, one of the main component of the Mediterranean diet, which has a polyphenolic profile comparable to the oil itself, but in greater quantities per unit of weight [14,15].

Precisely polyphenols are among the most important and studied bioactive molecules for their multiple beneficial properties primarily antioxidant and anti-inflammatory but also antibacterial and antiparasitic, as well as, for example, for their ability to regulate lipid levels and antiaging activity, for their positive effects on the intestinal microbiota, for their protective effect against metabolic syndrome and cardiovascular risk, for the treatment of type II diabetes and chronic degenerative diseases, as well as for their potential anticancer effects [7,10,11,12]. In particular due to their antioxidant activity they are extensively studied, given that oxidative stress is the basis of multiple pathological conditions, including gastrointestinal disorders and cancer. It is no coincidence that the European Food Safety Authority (EFSA) released in 2011 some health claims specifically concerning olive oil polyphenols [16]. Moreover, a 2005 publication analyzed the structure of oleocanthal, which is comparable to ibuprofen, and this would help explain how it performs its anti-inflammatory action [17]. 

Furthermore, the study of the health effects of agrifood waste-derived substances can lead to assess the possibility of also obtaining them from fruits and vegetables that are usually not used for food purposes due to flavors and aromas that are not appreciated by the consumer [18]. 

The study of nutraceutical substances and their possible biological effects and uses certainly gives new impetus to the food and pharmaceutical sectors.

The processing and study of by-products from agrifood wastes for obtaining bioactive substances has the ultimate goal of developing products that are usable by consumers, such as new food products (functional foods), formulations, and food supplements [19,20]. For this reason, dedicated studies are necessary, also related to the marketing of the finished product.

The articles published in this Special Issue deal with many topics in this increasingly current field of research, underlining the need for investigations involving different skills. We also believe they provide crucial knowledge and suggestions for future insights.

We are deeply grateful to all the authors and reviewers for their immense contribution and to the editorial team and Assistant Editor for their valuable and constant support.
